# Vascular Health Triad in Humans With Hypertension—Not the Usual Suspects

**DOI:** 10.3389/fphys.2021.746278

**Published:** 2021-10-01

**Authors:** Sushant M. Ranadive, Gabrielle A. Dillon, Sara E. Mascone, Lacy M. Alexander

**Affiliations:** ^1^Department of Kinesiology, University of Maryland, College Park, College Park, MD, United States; ^2^Department of Kinesiology, The Pennsylvania State University, University Park, PA, United States; ^3^Center for Healthy Aging, The Pennsylvania State University, University Park, PA, United States

**Keywords:** blood pressure, endothelium, inflammation, oxidative stress, reactive oxygen species

## Abstract

Hypertension (HTN) affects more than one-third of the US population and remains the top risk factor for the development of cardiovascular disease (CVD). Identifying the underlying mechanisms for developing HTN are of critical importance because the risk of developing CVD doubles with ∼20 mmHg increase in systolic blood pressure (BP). Endothelial dysfunction, especially in the resistance arteries, is the primary site for initiation of sub-clinical HTN. Furthermore, inflammation and reactive oxygen and nitrogen species (ROS/RNS) not only influence the endothelium independently, but also have a synergistic influence on each other. Together, the interplay between inflammation, ROS and vascular dysfunction is referred to as the vascular health triad, and affects BP regulation in humans. While the interplay of the vascular health triad is well established, new underlying mechanistic targets are under investigation, including: Inducible nitric oxide synthase, hydrogen peroxide, hydrogen sulfide, nuclear factor kappa-light-chain-enhancer of activated B cells (NF-κB) and nuclear factor activated T cells. This review outlines the role of these *unusual suspects* in vascular health and function in humans. This review connects the dots using these *unusual suspects* underlying inflammation, ROS and vascular dysfunction especially in individuals at risk of or with diagnosed HTN based on novel studies performed in humans.

## Introduction

Cardiovascular disease (CVD) is the leading cause of morbidity and mortality worldwide, and the prevalence of CVD increases with aging (Virani et al., 2021). The increase in blood pressure (BP) or diagnosed hypertension (HTN) is widely accepted as the primary precursor to CVD; the risk of CVD is assumed to increase in a linear fashion as BP increases. In general, the risk of CVD doubles when there is an approximately 20 mmHg increase in systolic BP and 10 mmHg increase in diastolic BP (Lewington et al., 2002). Further, BP rises substantially as humans age (Guzik and Touyz, 2017); however, the rise in BP with age is linear among men but rises in a differential pattern among women. Sex differences in HTN and their underlying mechanisms have been reviewed in detail previously (Ji et al., 2020).

In 2017, the American College of Cardiology (ACC) and American Heart Association (AHA) redefined the classifications of HTN diagnosis (Whelton et al., 2018). In the revised categorizations, the BP threshold has been lowered for stage 1 HTN and prehypertension (now elevated BP). Stage 1 HTN is defined as resting systolic BP between 130 and 139 mmHg or diastolic BP between 80 and 89 mmHg, and elevated BP is now defined as systolic BP between 120 and 129 mmHg and diastolic BP below 80 mmHg. Elevated BP is a strong predictor of late life HTN and CVD (Whelton et al., 2018). The revised categorizations have drastically increased the prevalence of HTN to about 46% of Americans (Virani et al., 2021), and have highlighted the importance of preclinical and clinical research in humans to identify therapeutic targets for interventions that extend the health of aging humans (Whelton et al., 2018). Moreover, identification of novel drug treatment based targets is important as medication adherence with traditional antihypertensives (diuretics, angiotensin converting enzyme inhibitors, calcium channel blockers, etc.) remains a significant issue.

**FIGURE 1:**
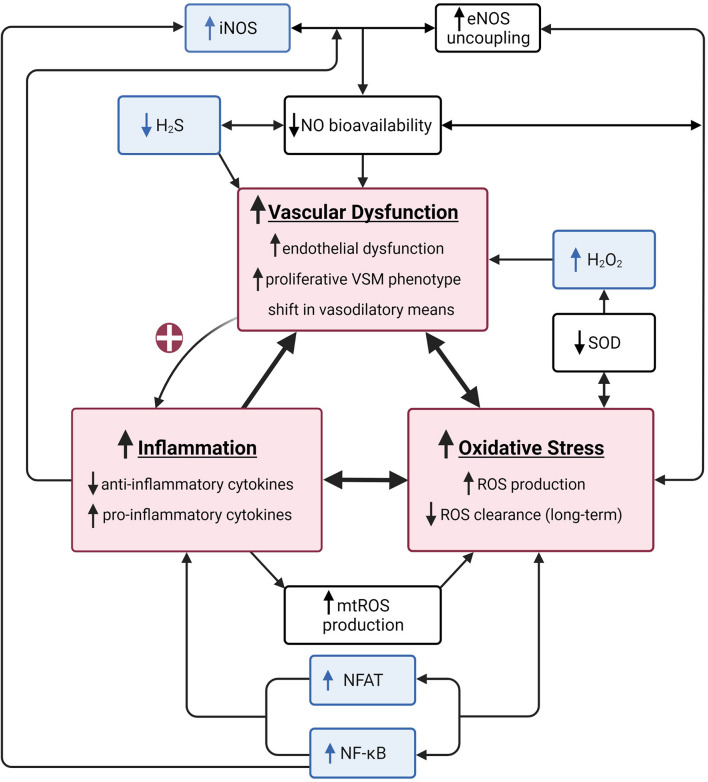
A summary of the vascular health triad. The vascular health triad is composed of oxidative stress, inflammation, and vascular dysfunction (red boxes). These outcomes are synergistically interdependent as their underlying mechanisms directly (e.g., high inflammatory state causes a decrease in nitric oxide (NO) bioavailability resulting in increased vascular dysfunction) or indirectly [e.g., vascular dysfunction increasing inflammation via positive feedback loop (red plus sign)] interact, resulting in a vicious cycle of increased cardiovascular disease risk. Well-established underlying mechanisms of the triad include decreased NO bioavailability via increased endothelial nitric oxide synthase (eNOS) uncoupling, decreased superoxide dismutase (SOD), and increased mitochondrial reactive oxygen species (mtROS) production (white boxes). The unusual suspects include inducible nitric oxide synthase (iNOS), hydrogen sulfide (H2S), hydrogen peroxide (H2O2), nuclear factor kappa-light-chain-enhancer of active B cells (NF-κB), and nuclear factor of activated T cells (NFAT; blue boxes). Created with BioRender.com.

The main site for vascular resistance, and thus a critical component of BP regulation, are the arterioles in the microcirculation (Nowroozpoor et al., 2021). The arterioles have similar anatomical layers as larger arteries; however, the lumen size of the arterioles (10–150 μm diameter) creates a substantial resistance to the blood flow and thus BP responses. In addition to the lumen size, the smooth muscle tone being normally in a state of contraction makes the resistance vessels an important site for BP control. Vascular resistance within the arteries is controlled by a complex interplay between local vasodilators, sympathetic modulation, and endocrine (paracrine) driven changes. Not only is the microvascular bed the first site to present with dysfunction, but it can also experience dysfunction without displaying any evidence in the macrovasculature or feed arteries (Nowroozpoor et al., 2021). Well established causes of endothelial dysfunction include imbalances in inflammation and/or reactive oxygen species (ROS). This is of particular importance because endothelial dysfunction can further potentiate imbalances in inflammation and ROS, leading to a “never ending cycle (or triad)” (Figure 1). The cumulative effects of these minor insults on the vasculature lead to a pro-hypertensive environment. In this mini-review we will first present aspects of the vascular health triad and then novel mechanisms that induce imbalances within the system, leading to HTN in humans. Table 1 summarizes the methods utilized to elucidate mechanisms underlying hypertension-associated vascular dysfunction A comprehensive discussion of the renal and sympathetic contributions to HTN is beyond the scope of this review, but have been recently reviewed (Grassi et al., 2018, 2019; Fonkoue et al., 2019; Holwerda et al., 2019; Keir et al., 2020).

**TABLE 1:** Summary of methodology for mechanisms mediating vascular function in hypertensive adults.

Method	References	Pathway
***In vivo***		
**Flow-mediated Dilation + Doppler Ultrasound**		
	Gokce et al., 2001; Benjamin et al., 2004; Juonala et al., 2004; McGowan et al., 2006; Buus et al., 2007; Yang et al., 2010; Broxterman et al., 2019; Figueiredo et al., 2012	Endothelium-dependent dilation
	Ratchford et al., 2019a	Endothelium-dependent dilation
		Oxidative stress (acute antioxidant cocktail)
	Sapp et al., 2021	Endothelium-dependent dilation
		Inflammation (influenza vaccine)
	Craighead et al., 2020	Endothelium-dependent dilation, Endothelium in-dependent dilation (sublingual NTG), Oxidative stress (Vitamin C)
**Venous Occlusion Plethysmography**		
	On et al., 2002	Endothelium-dependent (ACh)
	Bottino et al., 2015	Endothelium-dependent dilation, Endothelium in-dependent (sublingual NTG)
		Endothelium-dependent (ACh, Bradykinin)
	Hingorani et al., 2000	Endothelium in-dependent (NTG, verapamil)
		Inflammation (salmonella typhi vaccine)
**Arterial Infusions + Doppler Ultrasound**		
	Takase et al., 2006; Broxterman et al., 2019	Endothelium-dependent dilation (ACh)
	Gokce et al., 2001; McGowan et al., 2006; Buus et al., 2007	Endothelium in-dependent dilation (NTG)
**Iontophoresis + Laser Doppler Flowmetry**			
	Cupisti et al., 2000	Endothelium-dependent dilation (ACh) Endothelium-independent dilation (SNP)
	Farkas et al., 2004	
	Lindstedt et al., 2006	Endothelium-dependent dilation (ACh; local heating) Endothelium in-dependent dilation (SNP)
**Intradermal Microdialysis + Laser Doppler Flowmetry**		
	Smith et al., 2011	Endothelium-dependent dilation (ACh, local heating) NO (L-NAME) iNOS (1400W) nNOS (NPLA)
	Smith et al., 2013	Vasoconstriction (local cooling, yohimbine + proprananol), Rho/Rho-Kinase (fasudil)
	Bruning et al., 2015	Endothelium-dependent dilation (local heating), NO (L-NAME)
	Craighead et al., 2017	Endothelium-dependent dilation (ACh, local heating), NO (L-NAME), iNOS (1400w)
	HTN Greaney et al., 2017	Endothelium-dependent dilation (ACh), NO (L-NAME), H_2_S (Na2S, AOAA)
	Craighead et al., 2018	Endothelium independent dilation (SNP), NE-induced vasoconstriction, Lysyl Oxidation (BAPN)
	Dillon et al., 2020	Endothelium-dependent (ACh), NO (L-NAME)
**Sublingual NTG + Doppler Ultrasound**		
	McGowan et al., 2006	Endothelium in-dependent dilation
**Non-invasive Single Limb Exercise + Doppler Ultrasound**		
	Ratchford et al., 2019b	BF response to exercise (IHG)
	McGowan et al., 2006	BF response to exercise (static IHG, dynamic knee extension)
**Single Limb Exercise + NIRS-derived TSI**		
	Sprick et al., 2019	BF response to exercise (IHG)
***Ex vivo / in vitro* Circulating concentrations**		
	Hingorani et al., 2000	IL-1*β*, IL-1Ra IL-6, TNF-*α*
	Bottino et al., 2015	IL-1, IL-6, OxLDL, sICAM, sVCAM, sE-Selectin, TNF-*α*
	Junqueira et al., 2018	Adiponectin, CRP, endothelin, ICAM, VCAM
	Craighead et al., 2020	IL-6, OxLDL, TNF-*α*, total antioxidant status, hsCRP
**PBMCs**		
	Huang et al., 2016	IL-6, NFAT, TNF-*α*
**Arterial Biopsy**		
	Phillips et al., 2007	NO (L-NAME)
		Oxidative stress (H_2_O_2_)
	Donato et al., 2007	Oxidative stress (nitrotyrosine, NFκB)
	Migrino et al., 2011	Endothelial-dependent dilation (ACh), Endothelial in-dependent dilation (papaverine), Oxidative stress (SOD, BH4, mitoquinone, gp91ds-tat)
	Beyer et al., 2014	Oxidative stress (H_2_O_2_, mtROS, tempol)
	Beyer et al., 2017	Oxidative stress (H_2_O_2_, mtROS)
	Kadlec et al., 2017	H_2_O_2_, NO, PGC-1*α*
	Hughes et al., 2021b	NO (L-NAME, c-PTIO), H_2_O_2_ (peg-cat) Oxidative stress (rotenone)
	Hader et al., 2019	
	Hughes et al., 2021a	H_2_O_2_
**Venous Endothelial Cell Biopsy**		
	Donato et al., 2007	Oxidative stress (NADPH oxidase p47phox, SOD, NFκB)
	Pierce et al., 2009	Inflammation (NFκB, TNF-*α*)
		Oxidative stress (NADPH oxidase p47phox)
	Craighead et al., 2020	Oxidative stress (NADPH, MnSOD), Inflammation (NFkB)
**Cutaneous Biopsies**		
	Smith et al., 2011	eNOS, iNOS, nNOS, pVASP
	Smith et al., 2013	ROCK activity and expression
	HTN Greaney et al., 2017	H_2_S (CSE, 3-MPST)
**Myograph**		
	Buus et al., 2007	“small artery relaxation with ACh”

*3-MPST, 3-mercaptopyruvate sulfurtransferase; ACh, acetylcholine; BAPN, β-aminopropionitrile; CRP, C-reactive protein; CSE, cystathionine gamma-lyase; H_2_S, hydrogen sulfide; hsCRP, high-sensitivity C-reactive protein; IHG, intermittent handgrip; IL, interleukin; iNOS, inducible nitric oxide synthase; L-NAME, N^*G*^-nitro-l-arginine methyl ester; MnSOD, manganese superoxide dismutase; NADPH, nicotinamide adenine dinucleotide phosphate; NE, norepinephrine; NFkB, nuclear factor kappa-light-chain-enhancer of activated B cells; nNOS, neuronal nitric oxide synthase; NO, nitric oxide; NPLA, N(ω)-propyl-L-arginine; NTG, nitroglycerin; oxLDL, oxidized low-density lipoprotein; PBMCs, peripheral blood mononuclear cells; ROCK, rho-associated protein kinase; ROS, reactive oxygen species; sICAM, soluble inter-cellular adhesion molecule; SNP, sodium nitroprusside; sVCAM, soluble vascular cell adhesion molecule; TNF-α, tumor necrosis factor alpha.*

## Vascular Health—Role of Inflammation and Reactive Oxygen and Nitrogen Species*—“The Usual Suspects”*

In young, healthy adults, inflammation and reactive oxygen and nitrogen species (ROS/RNS) serve a critical, positive physiological role in vascular homeostasis. The maintenance of vascular health is the complex relationship between vasoprotective factors, such as the nitric oxide system, and other pathways that impair these mechanisms, including both inflammation and ROS. “Normal” vascular function is often characterized by the ability to efficiently vasodilate or vasoconstrict in response to a stimulus. On the contrary, vascular dysfunction is characterized by the loss of efficiency in the vasodilatory component even in the presence of a stimulus. *Inflammation* is one such stimulus (and a cornerstone of the vascular health triad) which influences vascular function acutely and chronically—in a temporal fashion. Inflammation is heightened as a natural defense mechanism to tissue injury, infection, or pathogen infiltration. The initial inflammatory cascade is characterized by heightened pro-inflammatory cytokine release, immune cell movement to the site of invasion or injury, and the release of local chemoattractants, notably, adhesion molecules (Wadley et al., 2013). This acute inflammation (influx of inflammatory cytokines) can transiently impair vascular function (8–32 h), through an acute impairment in nitric oxide (NO) bioavailability, even in young otherwise healthy individuals (Hingorani et al., 2000). The second component of the vascular health triad is ROS, which are free radicals, such as superoxide or peroxynitrite, that are integral to cellular signaling (El Assar et al., 2013; Wadley et al., 2013). ROS are produced via oxidative metabolism and proteins, such as NADPH oxidase, xanthine oxidase and via endothelial nitric oxide synthase (eNOS) uncoupling (Jacobi et al., 2005; Ding et al., 2007; El Assar et al., 2013; Wadley et al., 2013). Endogenous antioxidants, such as superoxide dismutase (SOD), glutathione, and NO, clear ROS enzymatically, or through direct chemical reaction (Selemidis et al., 2007; El Assar et al., 2013; Wadley et al., 2013). The aspect of mitochondrial ROS is covered in detail in an excellent review by Kirkman et al. (2021).

In young, otherwise healthy adults, there is ***(I)*** redundancy in the vasodilatory pathways with abundant bioavailability of endothelium-derived vasodilating substances (e.g., NO, prostaglandins, EDHF) ***(II)*** low basal concentrations of vasoconstrictive substances and ***(III)*** low basal concentrations of inflammatory cytokines and ROS. However, while there is ample data indicating impaired endothelial function in human subject cohorts who are at risk for the development of HTN later in life, including those with a family history of HTN (Greaney et al., 2015; Matthews et al., 2017), and non-traditional CVD risk factors (Martens et al., 2016; Greaney et al., 2019; Katulka et al., 2019), there is lack of data in individuals who are young and otherwise healthy but have undiagnosed HTN. Moreover, due to this redundancy it is difficult to unravel these interrelated mechanisms using gross measures of endothelial function in conduit arteries.

Our laboratory recently induced acute inflammation using an influenza vaccine *in vivo* and *in vitro* in young, healthy African American and Caucasian American individuals (Sapp et al., 2021). Although the vaccine stimulus did not impact conduit artery function (as measured by flow-mediated dilation), there were decreases in eNOS messenger RNA in the African American group, coinciding with race-specific changes in intracellular and extracellular microRNAs (miR) related to inflammation (miR-221-3p, 222-3p, and 150-5p) (Sapp et al., 2021). Circulating miRs are novel biomarkers of acute and chronic inflammation (Mi et al., 2013; Benz et al., 2016). miRs play mechanistic roles in endothelial activation, inflammation, and dysfunction; thus, initiating events in HTN (Cheng et al., 2014; Shi et al., 2015; Fernández-Hernando and Suárez, 2018). Furthermore, certain miRs [miR-146-protective (Wang et al., 2017); miR-34a- pro-inflammatory (Badi et al., 2018); miR-570-3p- pro-senescence (Baker et al., 2019); miR-217- pro-inflammatory (Zhang et al., 2019)] regulate ROS mechanisms via sirtuin-1 (Yamakuchi, 2012; Yamakuchi and Hashiguchi, 2018). Circulating miRs are taken up by endothelial cells, where they affect endothelial cell function, promote inflammation, and increase ROS (Njock and Fish, 2017; Zhong et al., 2018). Furthermore, the flow patterns within the arterial tree, i.e., turbulent flow near bifurcations or atherosclerotic lesions which can accentuate endothelial permeability or in contrast laminar flow which can exert beneficial effects on the vessel wall tend to trigger a number of miRs (Marin et al., 2013; Schmitz et al., 2019). Case in point, laminar shear stress (could be exercise induced) can upregulate miR-126 which is generally accepted as an anti-inflammatory and anti-atherogenic miR (Sapp et al., 2017). Therefore, in recent years circulating miRs have emerged as novel molecules mediating cell-to-cell communication in physiological processes (Njock and Fish, 2017; Bär et al., 2019). Mechanistically, emerging data in humans indicates significant cross-talk between inflammation and ROS within the endothelial cells in young, otherwise healthy adults, even before measurable conduit artery or macrovascular dysfunction plausibly through miRs (Sapp et al., 2021).

## Vascular Health Triad—Aging or Diseased States

In aging and diseased states, the vascular health triad becomes a positive feedback loop of heightened inflammation, oxidative stress, and vascular dysfunction. Chronic overproduction of inflammatory mediators, such as inflammatory cytokines, adhesion molecules, and inflammatory proteins, result in systemic low-grade inflammation initiating the complex cascade of heightened NADPH oxidase activity, eNOS uncoupling, and mitochondrial dysfunction, all of which result in heightened ROS production. Early in aging or disease pathogenesis, heightened antioxidant concentrations accommodate excessive ROS production. Following long-term exposure to increased ROS and inflammation, antioxidant defense mechanisms are reduced. Previous studies have interrogated these mechanisms with the acute administration of antioxidant cocktails (including ascorbic acid and alpha-tocopherol) in older adults (Eskurza et al., 2004a,b; Crecelius et al., 2010; Wray et al., 2012; Richards et al., 2015; Trinity et al., 2016), post-menopausal females (Ozemek et al., 2016), and individuals with heart failure with preserved ejection fraction (Ratchford et al., 2019a). However, more recent studies have focused on targeted pharmacological agents to identify specific ROS molecules within the triad (Alexander et al., 2013; Hurr et al., 2018; Martens et al., 2018; Park et al., 2018; Rossman et al., 2018). One emerging link in this triad is through the inducible nitric oxide synthase (iNOS) pathway. iNOS activity is increased during inflammation and NO is produced in toxic concentrations to prevent cell death by clearing excessive ROS (Aktan, 2004). NO reacts with superoxide ($O_{2}^{-}$), a main type of ROS produced during heightened inflammation, to form peroxynitrite (*ONOO*^−^). Concurrently, superoxide dismutase (SOD) reacts with superoxide ($O_{2}^{-}$) to form hydrogen peroxide (*H*_2_*O*_2_). Interestingly H_2_O_2_ becomes the main contributing vasodilatory substance when NO bioavailability is decreased (Phillips et al., 2007; Beyer et al., 2014; Kadlec et al., 2017). Initially with acute inflammatory/ROS stimuli, other vasodilatory substances, such as prostaglandins and H_2_O_2_, can compensate for the impairment in NO-mediated vasodilation (Beyer et al., 2017). However, if the allostatic load presented by inflammation and ROS persists, not only do the compensatory mechanisms fail, but eventually deleterious vascular remodeling occurs. Therefore, with aging and/or disease progression the healthy functioning of the vasculature is disrupted due to ***(I)*** loss of redundant vasodilatory pathways, with limited bioavailability of vasodilating substances (especially NO), ***(II)*** increased circulating vasoconstrictive substances and ***(III)*** increased concentrations of inflammatory cytokines and ROS.

Typically, when considering vascular dysfunction, the role of NO bioavailability/scavenging due to eNOS uncoupling, decreased superoxide dismutase (SOD), and increased mitochondrial reactive oxygen species (mtROS) production are of significant importance (Figure 1). Therefore, in the following sections we have presented some of the newer and not widely discussed factors which can also have role in decreased NO bioavailability and/or increased NO scavenging, leading to vascular dysfunction.

## Vascular Health Triad—“*The Unusual Suspects”*

### Role of Inducible Nitric Oxide Synthase

During heightened inflammation and oxidative stress, there are various intersecting pathways that contribute to impaired NO bioavailability and, eventually, vascular dysfunction. Increased iNOS expression is stimulated by nuclear factor kappa-light-chain-enhancer of activated B cells (NF-κB), interleukin-6 (IL-6), and ROS producers, such as the p47^*phox*^ subunit of NADPH oxidases (Hemmrich et al., 2003; Wu et al., 2008; Li et al., 2015). With chronic low-grade inflammation, iNOS activity increases, resulting in increased iNOS-derived NO production. To pharmacodissect the NOS balance in humans and prominent role of iNOS, we performed a unique *bed to bench* experiment evaluating endothelial-dependent microvascular function in individuals with HTN during iNOS, neuronal NOS (nNOS) and non-selective-NOS inhibition (Smith et al., 2011). Interestingly, the attenuated endothelial-dependent vasodilation in hypertensive adults was restored with iNOS inhibition, suggesting a prominent role of iNOS in hypertension-induced microvascular dysfunction. Even though the eNOS expression was similar between normotensives and hypertensives adults, iNOS expression in biopsy samples from hypertensive subjects was significantly greater as compared to age-matched normotensives (Smith et al., 2011). Thus, the NO produced during inflammation with HTN adopts a scavenging and cell-preserving role, as opposed to an active vasodilatory role.

### Hydrogen Peroxide (H_2_O_2_) and H_2_O_2_-Mediated Primary Vasodilatory Mechanism

SOD is a primary antioxidant defense system with three known forms: SOD1 (cytosolic), SOD2 (mitochondrial), and SOD3 (circulating) (Zelko et al., 2002). SOD scavenges O_2_^–^ radicals to form *H*_2_*O*_2_. During states of heightened inflammation and ROS, NADPH oxidase activity and eNOS uncoupling subsequently increase, resulting in increased O_2_^–^ production (El Assar et al., 2013; Risbano and Gladwin, 2013; Wadley et al., 2013). In early disease initiation and progression, SOD activity increases to clear excessively produced O_2_^–^, subsequently increasing the production of *H*_2_*O*_2_ during both acute and chronic increases in inflammation and oxidative stress (El Assar et al., 2013). Recent studies from the Gutterman laboratory have suggested a novel theory of shifts in vasodilatory pathways during vascular inflammatory conditions, especially HTN (Migrino et al., 2011; Beyer et al., 2014, 2017; Kadlec et al., 2017). The NO-mediated vasodilatory pathway is vasoprotective, as it helps maintain normal BP in young, healthy individuals. However, with increased intraluminal pressure, as seen in HTN and reduced bioavailability of NO, there is a shift in microvascular vasodilatory pathways toward H_2_O_2_-dependent mechanisms, even though the total magnitude of the vasodilation to a given shear stimulus remains the same (Beyer et al., 2014). Hughes et al. (2021b) examined the effect of transient increases in intraluminal pressure in resistance arterioles of hypertensive individuals with and without coronary artery disease were evaluated. In this model, there was a compensatory switch to the H_2_O_2_-mediated vasodilatory pathway following increased intraluminal pressure, suggesting diseased and healthy aged adults have similar shifts in primary vasodilatory mechanisms, but along a different time course (Hughes et al., 2021b). Furthermore, in the presence of transient increases in intraluminal pressure, even in isolated arterioles of healthy individuals, H_2_O_2_ is typically mitochondria derived (Beyer et al., 2014). These *ex-vivo* studies have also led to potential targets, such as autophagy and extranuclear telomerase (Hughes et al., 2021a). Taken together, these studies suggests an important switch in the physiological mechanism of vasodilation and heightened ROS production, specifically production of O_2_^–^, H_2_O_2_, and *ONOO*^−^as propagating a positive feedback loop that promotes vascular dysfunction.

### Nuclear Factor Kappa-Light-Chain-Enhancer of Activated B Cells and Nuclear Factor From Activated T Cells

Investigating upstream transcriptional molecular targets underlying endothelial and vascular smooth muscle cell dysfunction in humans has yielded significant understanding of the complexity of these signaling mechanisms. Despite the complexity and multiple downstream effects, these studies are necessary for the development of targeted treatment and prevention strategies for HTN in humans. It is well established that NF-κB are an important intracellular mediator of inflammation and vascular dysfunction (specifically, NO-mediated mechanisms) (Donato et al., 2007, 2009; Pierce et al., 2009; Lee et al., 2014). In an elegant series of preclinical and clinical studies, Donato et al. (2009) established that activation of NF-κB mediates age-related vascular dysfunction. These studies interrogated NF-κB functionality in human subjects using a short-term (4 days) high-dose oral salsalate approach (Pierce et al., 2009). Salsalate, a non-acytlated salicylate, inhibits NF-κB translocation to the nucleus, reducing ROS synthesis through NADPH oxidases (Kopp and Ghosh, 1994; Pierce et al., 1996). They demonstrated that oral salsalate reduces endothelial cell NF-κ p65 expression by ∼25%, total nitrotrysine, a global marker of oxidative stress, and NADPH oxidase p47^*phox*^ expression by 25 and 30%, respectively. Importantly, inhibition of NF-κB functionally resulted in improved NO-dependent vasodilation. Additionally, when comparing inactive to habitually active older adults, active adults had reduced NF-κB p65 expression, reduced nitrotyrosine, and endothelial function similar to their younger counterparts (Walker et al., 2014).

A growing body of literature suggests that the family of Ca^2+^/calcineurin-sensitive transcriptional factors of nuclear factor from activated T-cells (NFAT) may play an essential role as a molecular switch that initiates dysfunction in both the endothelium (Cockerill et al., 1995; Boss et al., 1998; Armesilla et al., 1999; Bochkov et al., 2002; Gonzalez Bosc et al., 2004; Zetterqvist et al., 2015; Huang et al., 2016) and vascular smooth muscle (Suzuki et al., 2002; Lipskaia et al., 2003; Amberg et al., 2004; Nilsson et al., 2006; Nieves-Cintrón et al., 2007; Orr et al., 2009; Pang and Sun, 2009; Nilsson-Berglund et al., 2010; Berglund et al., 2012; Shiny et al., 2016; Soudani et al., 2016; Govatati et al., 2019). NFATs regulate multiple downstream mechanisms that initiate vascular dysfunction. Specifically, NFATs ***(I)*** impair endothelial function through NO-dependent mechanisms (Armesilla et al., 1999; Bochkov et al., 2002; Johnson et al., 2003; Norata et al., 2007; Garcia-Vaz et al., 2020; Wang et al., 2020), ***(II)*** increase the expression of inflammatory mediators in the arterial wall promoting atherosclerosis (Pierce et al., 1996; Pang and Sun, 2009; Nilsson-Berglund et al., 2010; Berglund et al., 2012; Zetterqvist et al., 2014; Weng et al., 2017), and ***(III)*** initiate pathogenic VSM proliferation (Suzuki et al., 2002; Lipskaia et al., 2003; Nilsson et al., 2006; Nieves-Cintrón et al., 2007; Donato et al., 2009; Orr et al., 2009; Pang and Sun, 2009; Nilsson-Berglund et al., 2010; Yan et al., 2015; Shiny et al., 2016; Soudani et al., 2016; Govatati et al., 2019). In preclinical models, inhibition of NFAT has prevented the activation of inflammatory cytokines (Kiani et al., 2000; Zetterqvist et al., 2014; Bretz et al., 2015; Huang et al., 2016), enhanced eNOS expression (Smith et al., 2011), increased NO bioavailability (Friedman et al., 2014; Zetterqvist et al., 2014, 2015; Garcia-Vaz et al., 2020), prevented VSM proliferation (Lipskaia et al., 2003; Berglund et al., 2012; Shiny et al., 2016), lowered BP (Garcia-Vaz et al., 2020), and reduced total atherosclerotic load (Norata et al., 2007; Nilsson-Berglund et al., 2010; Zetterqvist et al., 2014). Currently, these investigations/findings are limited to cellular and animal models. There are promising approaches, including examining NFATs in the skin microcirculation and in peripheral blood mononuclear cells, for investigating the role of NFATs in humans (Huang et al., 2016). Elucidation of the role of NFATs and their putative upstream contributions to the vascular health triad in humans is still needed.

### Hydrogen Sulfide

As one of the three gasotransmitters ubiquitously synthesized in mammalian systems, hydrogen sulfide (H_2_S) is emerging as a critical component of vascular homeostasis (Polhemus and Lefer, 2014). HTN-associated microvascular dysfunction is characterized by a loss of endothelium-dependent signaling pathways, including hydrogen sulfide (H_2_S) (Cupisti et al., 2000; Lindstedt et al., 2006; Smith et al., 2011; Craighead et al., 2017; Greaney et al., 2017). Similar to NO, H_2_S exerts several beneficial physiological effects, including inhibiting inflammatory markers and leukocyte adhesion molecules, enhancing anti-inflammatory markers, and acting as an antioxidant (Polhemus and Lefer, 2014). NO and H_2_S vasodilatory pathways are synergistically interdependent. Both exogenous and endogenous enzymatic H_2_S synthesis helps to maintain NO bioavailability by stabilizing the eNOS dimer and improving tetrahydrobiopterin (BH_4_) bioavailability (Zhao et al., 2001; Coletta et al., 2012; Altaany et al., 2013; Polhemus and Lefer, 2014; Greaney et al., 2017). Exogenous administration of H_2_S improves NO-dependent vasodilation, while blockade of the H_2_S producing enzyme, cystahionine y-layase (CSE), impairs NO-dependent vasodilation (Coletta et al., 2012; Altaany et al., 2013). Reciprocally, blockade of NO synthesis also reduces H_2_S-dependent vasodilation (Zhao et al., 2001; Coletta et al., 2012). We reported that the H_2_S-dependent contribution to endothelium-dependent vasodilation is functionally absent in naïve-to-therapy hypertensive adults (Greaney et al., 2017). This was partially due to reduced endogenous enzymatic synthesis of H_2_S, as expression and activity of H_2_S producing enzymes, including cystathione-γ-lyase and 3-mercaptopyruvate transulferase, were reduced in hypertensive compared to normotensive adults (Greaney et al., 2017). However, vascular responsiveness to exogenous H_2_S donors remained intact in hypertensive adults. In preclinical models, treatment with an H_2_S donating antihypertensive agents improves endothelial function and normalizes BP (Bucci et al., 2014; Ji et al., 2014; Al-Magableh et al., 2015; Xue et al., 2015). These improvements were partially mediated by increased NO bioavailability (Bucci et al., 2014; Ji et al., 2014; Al-Magableh et al., 2015; Xue et al., 2015), demonstrating the synergistic nature of these gasotransmitter pathways. Various nutraceutical intervention studies suggest that allicin, the bioactive component of garlic, improves vascular function, specifically through the H_2_S enzymatic pathway (Cui et al., 2020). Due to the ubiquitous nature of H_2_S, there are several clinical trials evaluating the impact of H_2_S donating pharmacologics in a variety of disease states. At present, the H_2_S synthetic pathway remains an underexplored therapeutic target in human HTN and other CVDs, including heart failure.

#### Potential Targets

In humans, the potential targets to mitigate the vascular health triad and improve the vascular function have been pursued from a global, holistic approach of exercise training to more specific, targeted treatments. In humans, ascorbic acid and folic acid supplementation have been shown to improve vascular function in populations with inflammatory diseases (Alexander et al., 2013; Karbach et al., 2014; Stanhewicz et al., 2015; Stanhewicz and Kenney, 2017). BH_4_ precursors and antioxidants are the main therapeutic targets facilitating eNOS coupling and reducing eNOS-derived ROS production. BH_4_ in the form of saproterin is an orphan drug used in the treatment of certain genetic variants of phenylketonuria. Saproterin has improved eNOS function and NO-dependent vasodilation in aged and hypercholesterolemic human subjects in both acute and interventional studies. Specifically, saproterin (or, BH_4_) supplementation works through eNOS coupling mechanisms and not simply through its moderate antioxidant capacity. Folic acid (and its active metabolite 5-MTHF) is a cost-effective strategy for improving BH_4_ bioavailability through BH2 recycling. Thus, reducing eNOS uncoupling is an attractive, accessible, and affordable intervention for improving the vascular health triad in aging and diseased states.

Similarly, mitochondrial ROS targeted interventions have shown promise in the past decade. Nicotinamide riboside (NR) is a sirtuin-1 (SIRT-1) precursor that has gained recent popularity in vascular intervention treatment research. NR is a precursor to NAD^+^, and SIRT1 is a NAD^+^-dependent deacetylase. Similar to resveratrol and Mito Q, NR supplementation has had vastly positive impacts on vascular function and oxidative stress in pre-clinical animal models (Yoshino et al., 2011; Mills et al., 2016). NR has been shown to improve vascular function and reduce oxidative stress in aged mice. However, in humans, these findings have not been replicated. NR has shown small impacts on various measures of vascular function (Martens et al., 2018). Thus, there is a knowledge gap in the positive benefits of long-term NR supplementation.

## Conclusion

In summary, there is ample evidence that the shift from healthy endothelial function to dysfunction, typically preceding HTN and CVD, is driven by a cross-talk between inflammation and ROS. There are numerous “players” that have been recently identified to be responsible for this abnormal shift toward dysfunction. Therefore, it is crucial to investigate these key players in human experimental models to further understand and improve human vascular function with the ultimate goal of preventing CVD risk.

## Author Contributions

SR and LA conceived the research review. SR, GD, SM, and LA designed the research review, drafted, revised, and approved the final version of the manuscript. GD and SM prepared figure and table. All authors contributed to the article and approved the submitted version.

## Conflict of Interest

The authors declare that the research was conducted in the absence of any commercial or financial relationships that could be construed as a potential conflict of interest.

## Publisher’s Note

All claims expressed in this article are solely those of the authors and do not necessarily represent those of their affiliated organizations, or those of the publisher, the editors and the reviewers. Any product that may be evaluated in this article, or claim that may be made by its manufacturer, is not guaranteed or endorsed by the publisher.
